# Surgery

**Published:** 1910-09

**Authors:** J. Lacy Firth


### SURGERY.

**Hypophysectomy** has now been performed a sufficient number of times for a rough estimate to be made of its usefulness as a remedy for certain disorders and of its difficulties and dangers. To assist in the formation of such an estimate, I have drawn up tables showing in brief outline the leading symptoms, pathology, operative technique, and results of operation in all those cases of hypophysectomy of which I have been able to find records in the library of the Bristol Medico-Chirurgical Society.

Broadly considered, the *indications* for the operation have been of the following three kinds :—

(1) The presence of symptoms and signs of pressure upon the optic chiasma.

(2) The presence of symptoms which are considered to show that the normal function of the anterior part of the hypophysis cerebri is disturbed.

(3) The presence of skiagraphic evidence that pressure changes have taken place in the sella turcica.

The indications of the second group are based upon the hypothesis that the syndrome of Pierre Marie, known as acromegaly, and also gigantism, are due to hyperpituitarism, and that the syndrome of Fröhlich, in which increased adiposity, anomalies of hair development and arrested sexual activity or development (infantilism) are the characteristic symptoms, is due to hypopituitarism. The appended tables have been drawn up with recognition of these distinct types of case, following von Eiselsberg and other writers on the subject.

The evidence which can be adduced in support of the above hypotheses with regard to pituitarism is not, however, free from confusion, especially perhaps in the case of acromegaly. In that affection the hypophysis is said to be often unchanged, though perhaps the observations in such cases have been mostly macroscopical and not microscopical.

Conversely, in many cases of tumour of the hypophysis there have been no signs of acromegaly. In at least one case of acromegaly [1] the pituitary gland was found completely destroyed by cysts and induration, a state of things hardly likely to be associated with increased function. Kocher in a case of acromegaly removed a round-celled sarcoma from the hypophysis (No. VI in the table), with the result that the characteristic symptoms of acromegaly materially improved. He remarks upon the difficulty of believing that the presence of an extensive soft sarcoma in the gland could have increased its functional out-put, and suggests as a possible explanation of the phenomena that the tumour had an indirect effect upon the infundibular region of the brain, in which region Erdheim has assumed a

[1] *Vide* Kocher, *Deutsche Ztschr. f. Chir.*, 1909, c. 30.

trophic centre to exist. Kocher also alludes to the fact that adiposity has been observed in cerebellar affections and in affections of the corpora quadrigemina, associated with increased pressure in the third ventricle. On the other hand, Cushing's case (No. VII in the table) supports the view that acromegaly is an expression of hyperpituitarism, for the portion of the gland removed yielded microscopical evidence of activity, and no tumour was present.

Cushing,[1] in conjunction with S. J. Crowe and J. Homans, has removed the whole or portions of the hypophysis from more than a hundred dogs. He found that for prolonged life it was essential to leave at least a fragment of the gland, and that after partial removal the animals became excessively fat, and in every instance showed marked atrophy of the organs of generation.

The improvement of patients with the Fröhlich type of symptoms, adiposity with infantilism, after evacuating cysts or removing tumours of the hypophysis, is naturally explained by assuming that the portions of gland left, having been relieved from pressure, have resumed their normal function.

It is interesting to note in this connection that Marburg[2] has brought forward facts which seem to show that the pineal gland is related to adiposity and sexual potency in the opposite sense to the pituitary gland, hypopinealism being associated with genital hypertrophy and hyperpinealism with adiposity.

The appended tables contain a brief summary of the facts concerning fourteen cases of trans-sphenoidal hypophysectomy. Two additional cases in which Cushing operated and two—one successful—in which Halsted (Chicago) operated are not included, the records being inaccessible. Also there have been operations of the kind, by Sir V. Horsley and others, by other routes. It will be noted that of the tabulated cases von Eiselsberg performed the operation in six, Hochenegg in three, and the other surgeons each in one case. The first case operated upon on the Continent was that of Schloffer in March, 1907.

*Operative Mortality.*—Reference to the column in the tables giving results will show that four of the fourteen patients died directly in consequence of the operation. This is a mortality of $28\frac{1}{7}$ per cent. The causes of death were meningitis in two cases, status lymphaticus, etc., in one case, and aspiration-pneumonia in one. There had been much bleeding at the operation in the last case. Case XI nearly ended in death, for the patient had violent meningitis at the end of a week. Obviously the operation is one which at present is attended with grave risk.

Hochenegg,[3] from the point of view of operative prognosis, divides cases of pituitary tumour into the following classes :—

[1] *Ann. Surg.*, 1909, l, 1002.
[2] *Vide* Kocher, *op. cit.*
[3] *Deutsche Ztschr. f. Chir.*, 1909, c. 317.

(1) Those in which there is a small tumour growing towards the sella and the sella is bridged over above by dura mater. In these there is a possibility of totally removing the tumour by the nasal route.

(2) Those in which the tumour fills the sella and also has a more or less widespread endocranial extension. In some of these cases it may be possible to relieve intracranial pressure by partial removal of the growth with improvement of vision and other symptoms; but in others, in which the greatest part of the tumour is in the brain above the sella, exposure of the tumour by the nasal route will be harmful, injuring the growth and setting up meningitis.

Unfortunately, it is impossible to tell beforehand which are the cases with extension and which are not. Case V is an instance in point. The patient was apparently in an early stage of the disease, was up and about and able to work, and yet the tumour had involved the frontal lobes extensively. Cases II and VI also belong to this group.

As Hochenegg points out, it is almost a matter of luck whether one operates upon a case belonging to the first or the second of the two classes described above, for the present methods of diagnosis do not enable one to distinguish the operable from the inoperable class, and improved technique will not diminish this difficulty. Possibly a preliminary exploration by the intracranial method would be the best in some doubtful cases.

*Results in the cases recovering from the operation.*—These are interesting and encouraging. The characteristic symptoms of acromegaly have been ameliorated, vision has improved, the hands, feet and head have become smaller, sometimes noticeably so a few days after the operation. In the cases of adiposo-genital degeneration also the symptoms have been abolished or materially relieved, and the improvement has been maintained for two or three years. On the other hand, two of the patients who recovered from the operation enjoyed their relief from certain symptoms but a short time, namely two and a half months and one month respectively (Nos. V and VI). The latter case was unsuitable for operation, as shown by the *post-mortem* examination, but not shown by diagnostic considerations beforehand.

*Pathology.*—The tumours, it will be seen, have been adenomata, carcinomata, sarcomata, or cysts. In Case VII there was no tumour. The duration of life and health after operation in some of the cases of sarcoma and carcinoma (see Cases IX, X and XIII) has been very remarkable, and it is remarkable, again, to find that in Cases II and VIII the patients were found to have malignant tumours, and yet they had been suffering from acromegaly eight or nine years. This long history suggests that the tumours had been of strangely slow growth (v. Eiselsberg).

*Technique of the Operation.*—The sphenoidal region has been reached through the upper part of the nasal cavities, with or without opening the frontal sinuses, or through the lower part of the nose by the method of Kanavel.[1] The sphenoidal sinus is opened, and through it the sella turcica, and the tumour excised with sharp spoons or other suitable instruments. The last-named method, that of Kanavel, seems the most promising. Von Eiselsberg, though he himself operated by deflecting the nose to one side and opening up the frontal sinuses, remarks[6] that having seen Cushing operate with technique similar to that of Kanavel, he is so favourably impressed that he has decided to give it a trial, though he thinks that temporary resection of the frontal sinuses walls, " giving, as it does, a larger access to the field of operation, must in certain cases be of decided advantage." The Kanavel technique is briefly outlined in Case XII (see table). As a prophylactic against meningitis from the operation Cushing[2] recommends the administration of urotropine beforehand. This recommendation is based upon S. J. Crowe's discovery that formaldehyde is rapidly excreted in the cerebro-spinal fluid.

J. LACY FIRTH.

TABLE I. EIGHT CASES OF HYPOPHYSECTOMY FOR ACROMEGALY (HYPERPITUITARISM).

| No. of Case. | Surgeon. | Date of Operation. | Age and Sex. | Duration of Symptoms. | Leading General Symptoms. | Ocular Symptoms. | Result of Radiography. | Pathology, etc. | Technique. | Results. |
|---|---|---|---|---|---|---|---|---|---|---|
| i. | Schloffer .. .. | March, 1907 | .. | .. | .. | .. | .. | .. | .. | R. Lived 2½ months. |
| ii. | Von Eiselsberg (*Ann. Surg.*, 1910, ii, 1) | Dec., 1907 | F. 33 | 8 years | Acromegaly, severe headache | Bi-temporal hemianopsia | .. | Basal sarcoma, extending to frontal lobes of brain | Lateral deflection of nose and removal of anterior wall of frontal sinuses | D. From meningitis 48 hours after operation. |
| iii. | Hochenegg .. (Case i.) (*Deutsche Ztschr. f. Chir.*, 1909, c. 317) | Feb., 1908 | .. | .. | Acromegaly | .. | .. | Tumour localised in the sella | Through upper part of nose and frontal sinus | C. Complete cure. Seen 2 years later. |
| iv. | Hochenegg .. (Case ii.) | .. | .. | .. | .. | .. | .. | .. | .. | R. |
| v. | Hochenegg .. (Case iii.) (*Op. cit.*) | Feb., 1909 | F. 33 | 3 years | Acromegaly, severe headaches, amenorrhœa | Right eye, narrowed field. Left eye, atrophy of left half of papilla and temporal hemianopsia | Enlarged sella | Malignant tumour of hypophysis, involving frontal lobes | Through upper part of nose, with deflection upwards of osteoplastic frontal flap | D. Death within 24 hours. Autopsy showed status lymphaticus, hydrocephalus internus, degeneration of myocardium, liver and kidneys. |

TABLE I (*continued*).

| [illegible]o. of [illegible]se | Surgeon. | Date of Operation. | Age and Sex. | Duration of Symptoms. | Leading General Symptoms. | Ocular Symptoms. | Result of Radiography. | Pathology, etc. | Technique. | Results. |
|---|---|---|---|---|---|---|---|---|---|---|
| [illegible]i. | Kocher .. .. (*Deutsche Ztschr. f. Chir.*, 1909, c. 13) | Jan., 1909 | F. 30 | 2 years | Acromegaly, headache, amenorrhœa | Choked discs, hemianopsia, central scotoma left side | Enlarged sella | Round-celled sarcoma invading the cavernous sinus and base of skull | Temporary splitting of upper part of nose in middle line, and osteoplastic deflection of flaps to each side. Frontal sinuses not opened | R. Abatement of all symptoms for a month, then rather sudden death from increased intra-cranial pressure. |
| [illegible]ii. | Harvey Cushing (*Ann. Surg.*, 1909, l. 1,002) | March, 1909 | M. 38 | 7 or 8 years | Acromegaly, headache | Photophobia, normal fields of vision | Slight enlargement of sella | No tumour. Glandular tissue showed signs of excessive activity | Frontal sinus walls and nasal bones turned down in osteo-plastic flap. Ethmoid cells removed | R. Ten weeks later headache absent, acromegalic appearance of face improved. |
| [illegible]iii. | Von Eiselsberg (*Op. cit.*) | March, 1910 | F. 40 | 9 years | Acromegaly, headache, insomnia | Normal fields.. | Enormous sella | Tumour pronounced to be parietal sarcoma by Stoerck, carcinoma of hypophysis by Erdheim | Osteo-plastic deflection of nose to right, frontal sinuses opened | D. Death from suppurative meningitis after three days. |

TABLE II. CASES OF HYPOPHYSECTOMY FOR ADIPOSO-GENITAL DEGENERATION (HYPOPITUITARISM).

| ›. f e. | Surgeon. | Date of Operation. | Age and Sex. | Duration of Symptoms. | Leading General Symptoms. | Ocular Symptoms. | Result of Radiography. | Pathology, etc. | Technique. | Results. |
|---|---|---|---|---|---|---|---|---|---|---|
| . | Von Eiselsberg (*Op. cit.*) | June, 1907 | M. 20 | About 7 years | Infantilism, headache | Right eye, atrophy of temporal half of papilla. Left eye, "genuine atrophy" | Enlargement of sella | Infiltrating growth of epithelial character, with cyst (pre-cancerous, Stoerck) | As in Cases ii and viii | R. Two years and nine months later adiposity decreased, vision much better, hair growing on scrotum. |
| . | Von Eiselsberg (*Op. cit.*) | Dec., 1907 | M. 27 | 3 years | Fatness, headache, loss of sexual power | Bi-temporal hemianopsia | Large excavation in position of sella | Angio-sarcoma of hypophysis | As in cases ii, viii and ix | R. Two years and three months later patient "doing well," vision much better. |
| i. | Von Eiselsberg (*Op. cit.*) | Dec., 1908 | F. 18 | 2 years | Vomiting, headache, fatness, infantilism | Right eye, vision one-third. Left eye, could only distinguish fingers. Bi-temporal hemianopsia | Absence of great part of sella | Cyst containing two teaspoonfuls of chocolate-brown fluid. No evidence of malignancy | As in Cases ii, viii, ix, etc. | C. The patient had meningitis after a week, but recovered. Two years later patient absolutely normal except for temporal hemianopsia in left side. Vision much improved. |

TABLE II (*continued*).

| No. of Case. | Surgeon. | Date of Operation. | Age and Sex. | Duration of Symptoms. | Leading General Symptoms. | Ocular Symptoms. | Result of Radiography. | Pathology, etc. | Technique. | Results. |
|---|---|---|---|---|---|---|---|---|---|---|
| xii. | S. J. Mixter and A. Quackenboss (*Ann. Surg.*, 1910, lii, 15) | Dec., 1909 | M. 27 | About 5 years | Infantilism | Bi-temporal hemianopsia, optic atrophy | Enlargement of sella | An ounce and a half of cholesterine-laden fluid escaped from a cyst | Method of Kanaval, viz. U-shaped incision beneath nose, nose turned up, bony-septum removed submucously, turbinates held back by retractors. | R. Four months later patient was "feeling fine." Vision much improved, temporal hemianopsia still present. |

TABLE III. HYPOPHYSECTOMY FOR CASES OF MIXED OR DOUBTFUL TYPE.

| No. of Case. | Surgeon. | Date of Operation. | Age and Sex. | Duration of Symptoms. | Leading General Symptoms. | Ocular Symptoms. | Result of Radiography. | Pathology, etc. | Technique. | Results. |
|---|---|---|---|---|---|---|---|---|---|---|
| iii. | Von Eiselsberg (*Op. cit.*) | Jan., 1909 | M. 36 | About a year | Intense headache, impotency | "Choked disc" in both eyes, temporal hemianopsia right side | Enlargement of sella | Large tumour of hypophysis, epithelial carcinoma. | As in Cases ii, etc. | R. Left eye remained blind. Fatness diminished, feet and hands became smaller. Head-circumference diminished. Signs of returning sexual power. Seen one year and three months later. |
| iv. | F. Smoler .. .. (*Wien. klin. Wchnschr.*, 1909, xxii, 1,488) | .. | .. | .. | .. | .. | Enlargement of sella | Cherry-sized tumour of hypophysis (adenoma) metastasis in cerebellum | Nasal route. | D. Death from aspiration pneumonia in 60 hours. |

